# Correcting Susceptibility Artifacts of MRI Sensors in Brain Scanning: A 3D Anatomy-Guided Deep Learning Approach

**DOI:** 10.3390/s21072314

**Published:** 2021-03-26

**Authors:** Soan T. M. Duong, Son Lam Phung, Abdesselam Bouzerdoum, Sui Paul Ang, Mark M. Schira

**Affiliations:** 1 School of Electrical, Computer and Telecommunications Engineering, University of Wollongong, Wollongong, NSW 2522, Australia; phung@uow.edu.au (S.L.P.); bouzer@uow.edu.au (A.B.); spa995@uowmail.edu.au (S.P.A.); 2 Applied Science Division, VinBrain, VinGroup, Hanoi 100000, Vietnam; 3 Faculty of Information Technology, Le Quy Don Technical University, Hanoi 122300, Vietnam; 4 ICT Division, College of Science and Engineering, Hamad Bin Khalifa University, Doha 34110, Qatar; 5 School of Psychology, University of Wollongong, Wollongong, NSW 2522, Australia; mschira@uow.edu.au

**Keywords:** susceptibility artifacts, deep learning, high-speed, echo planar imaging, reversed phase-encoding

## Abstract

Echo planar imaging (EPI), a fast magnetic resonance imaging technique, is a powerful tool in functional neuroimaging studies. However, susceptibility artifacts, which cause misinterpretations of brain functions, are unavoidable distortions in EPI. This paper proposes an end-to-end deep learning framework, named TS-Net, for susceptibility artifact correction (SAC) in a pair of 3D EPI images with reversed phase-encoding directions. The proposed TS-Net comprises a deep convolutional network to predict a displacement field in three dimensions to overcome the limitation of existing methods, which only estimate the displacement field along the dominant-distortion direction. In the training phase, anatomical T1-weighted images are leveraged to regularize the correction, but they are not required during the inference phase to make TS-Net more flexible for general use. The experimental results show that TS-Net achieves favorable accuracy and speed trade-off when compared with the state-of-the-art SAC methods, i.e., TOPUP, TISAC, and S-Net. The fast inference speed (less than a second) of TS-Net makes real-time SAC during EPI image acquisition feasible and accelerates the medical image-processing pipelines.

## 1. Introduction

Echo planar imaging is a fast magnetic resonance imaging (MRI) technique that has served as an important non-invasive tool in cognitive neuroscience [1]. EPI is widely used to record the functional magnetic resonance imaging (fMRI) data for studying human brain functions [2]. It is also the technique of choice to acquire the diffusion-weighted imaging (DWI) data for analyzing brain connection patterns [3]. Despite its popularity, EPI is prone to susceptibility artifacts (SAs) [4,5] and eddy-current artifacts [6,7], which consist of geometric distortions. The geometric distortions cause misalignments between the functional image and the underlying structural image, subsequently leading to errors in brain analysis, e.g. incorrect localization of neural activities in the functional brain studies. Therefore, an accurate geometric distortion correction method is crucial for applications that rely on EPI images.

In this study, we investigate the susceptibility artifact correction (SAC), as SAs are inevitable in EPI [5]. Interestingly, two EPI images, which are acquired using identical sequences but with reversed phase-encoding (PE) directions, have opposite patterns of geometric distortions caused by SAs [8,9]. Consequently, the middle version of the reversed-PE image pair is considered the distortion-free image. Chang and Fitzpatrick proposed to correct the SAs in two reversed-PE images by finding the corresponding points between two reversed-PE images; the corrected image was then formed by the mean intensity of the corresponding points [4]. Since displacements are estimated in lines along the PE direction independently, the estimated displacement field is not smooth, subsequently leading to unrealistic corrections. Andersson et al. proposed a method, called TOPUP, by modeling the displacement at each voxel as a function of discrete cosine basis functions [10]. This method estimates the entire *displacement field* along the PE direction, thereby avoiding the unsmooth problem.

Several reversed-PE based SAC methods have adopted an image registration approach, in which the corrected image is treated as the intermediate version of the two distorted input images. The two distorted reversed-PE images are transformed to the corrected image by an equal displacement amount but with the opposite directions. This registration approach for reversed-PE SAC was firstly proposed in [9]. Ruthotto et al. introduced a regularization term, inspired by the hyper-elastic registration, to constrain the displacement field in the registration framework, thereby achieving more realistic corrected images [11]. Hedouin et al. introduced the block-matching algorithm that estimates the displacement field at the block level of the given EPI image pair [12]. In another approach, Irfanoglu et al. introduced an anatomical regularizer based on the T2-weighted ($T_{2w}$) image to the registration framework so as to align better the corrected images to the underlying anatomical structure [13]. Duong et al. utilized T1-weighted ($T_{1w}$) for correction regularization, as the $T_{1w}$ images are routinely acquired in brain studies [14,15]; this method is called TISAC.

The above SAC methods require an iterative-optimization algorithm to estimate the displacement field and then compute the corrected images. This computation-intensive optimization step can take from 1 to 12 min, for an image pair of size 192 × 192 × 36 voxels [15]. Recently, Duong et al. proposed an end-to-end deep learning framework, called S-Net, to map a pair of 3D input reversed-PE images to a displacement field in the phase-encoding direction, and to provide the corrected image pair [16]. S-Net is trained using a set of reversed-PE image pairs. A new image pair is corrected by feeding the distorted image pair to the trained S-Net model directly, thereby reducing the processing time. The results of S-Net demonstrate the feasibility of using a deep network for the SAC problem. While providing a competitive correction accuracy, S-Net could still be improved in terms of correction accuracy, robustness to input image sizes, and imaging modalities.

To reduce computation time and increase robustness, existing SAC methods estimate the displacement field only along the phase-encoding direction (i.e., 1D distortion model). This is based on the fact that the distortions in the PE direction are prominent, whereas the distortions in the other directions are insignificant. In this study, we propose a generalized approach to enhance the correction accuracy by considering the distortions in all three directions (i.e., 3D distortion model). The 3D displacement field is predicted through a 3D convolutional encoder–decoder given a 3D reversed phase-encoding image pair. The convolutional network is trained end-to-end using the $T_{1w}$ modality as an auxiliary condition. The proposed method is called anatomy-guided deep learning SAC, or TS-Net in which the letter “T” arises from $T_{1w}$.

The new contributions of this paper are highlighted as follows:

1. We design a deep convolutional network to estimate the 3D displacement field. The deep network is designed to make TS-Net robust to different sizes, resolutions, and modalities of the input image by using batch normalization (BN) layers and size-normalized layers.
2. We estimate the displacement field in all three dimensions instead of only along the phase-encoding direction. In other words, TS-Net predicts the displacement field that captures the 3D displacements for every voxel. This, to our knowledge, is a significant improvement compared to most existing SAC methods [10,16], which estimate the distortions only along the PE direction and ignore the distortions along with the other two directions.
3. We introduce a learning method that leverages $T_{1w}$ images in the training of TS-Net. The motivation is that the $T_{1w}$ image is widely considered as a *gold standard* representation of a subject’s brain anatomy [17], and it is readily available in brain studies [18]. To make TS-Net more applicable for general use, the $T_{1w}$ image is used *only* in training for network regularization, but not in the inference phase.
4. We provide an extensive evaluation of the proposed TS-Net on four large public datasets from the Human Connectome Project (HCP) [19]. First, an ablation study is conducted to analyze the effects of using different similarity measures to train TS-Net, the effects of various components in the TS-Net framework, and the effects of using a pre-trained TS-Net when training a new dataset. Second, TS-Net is compared with three state-of-the-art SAC methods, i.e., TOPUP [10], TISAC [15], and S-Net [16], in terms of correction accuracy and processing time.

The remainder of this paper is organized as follows. Section 2 describes the materials and the proposed method. Section 3 presents the experimental results, and Section 4 discusses the proposed method and results. Finally, Section 5 summarizes our work.

## 2. Materials and Methods

In this section, Section 2.1 describes the EPI datasets used for experiments. Section 2.2 introduces the proposed TS-Net method. Section 2.3 presents the methods used for conducting experiments.

### 2.1. EPI Datasets

To evaluate the SAC methods, we used four EPI datasets (fMRI-3T, DWI-3T, fMRI-7T, and DWI-7T), which are the unprocessed data of the *Subjects with 7T MR Session* from the public Human Connectome Project repository. The functional and diffusion MRI datasets were used to study functional connectivity of the human brain and reconstruct the complex axonal fiber architecture, respectively [20,21]. These four datasets were acquired using different acquisition sequences, imaging modalities, field strengths, resolutions, and image sizes; thus, the datasets are diverse in size and distortion property. Table 1 shows a summary of the four datasets. Note that the apparent diffusion coefficient map was not acquired in the DWI datasets. The b-values were 1000, 2000, and 3000 s/mm${}^{2}$ for the DWI-3T dataset, and 1000 and 2000 s/mm${}^{2}$ for the DWI-7T dataset.

### 2.2. The Proposed TS-Net Method

**Figure 1:**
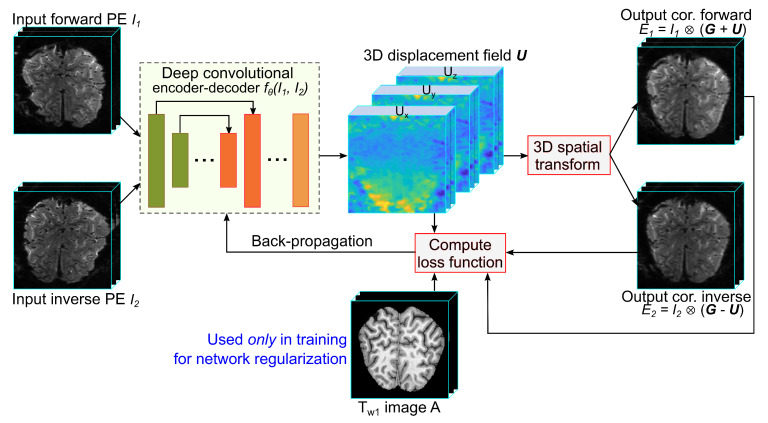
The proposed learning framework (TS-Net) for correcting the SAs in reversed-PE images. TS-Net accepts a pair of 3D reversed-PE images and produces the 3D displacement field and the corrected images.

This section introduces a 3D anatomy-guided deep learning framework, called TS-Net, to correct the susceptibility artifacts in a 3D reversed-PE image pair (see Figure 1). The proposed TS-Net includes a deep convolutional network to map the 3D image pair to the 3D displacement field U. It also has a 3D spatial transform unit to unwarp the input-distorted images with the predicted displacement field, providing the corrected images. In contrast to existing SAC methods [15,16], TS-Net estimates the 3D displacement field, or three displacement values for each voxel. Thus, the displacement field U can be represented as $\left[U_{x},U_{y},U_{z}\right]$, where $U_{d}$ is the displacement field in the *d* direction.

The 3D spatial transform unit is the interpolation operator to unwarp or resample the input images by the estimate displacement field [22]. Let U denote the displacement field of image $I_{1}$ to the corrected image, then −U is the displacement field of image $I_{2}$ to the corrected image because of the inverse distortion property of the reversed-PE image pair. The spatial transform unit produces the corrected images, expressed as $E_{1}=\left[I_{1}⊗\left(G+U\right)\right]$, and $E_{2}=\left[I_{2}⊗\left(G-U\right)\right]$, where ⊗ is the linear interpolation and $G=[G_{x},G_{y},G_{z}]$ is the regular grids in the *x*, *y*, and *z* directions.

**Figure 2:**
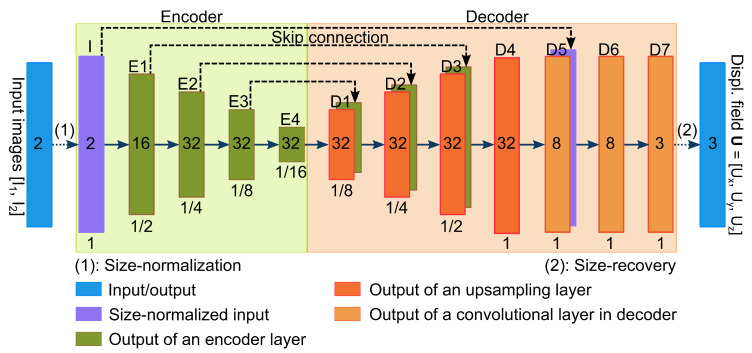
The convolutional encoder–decoder for mapping a pair of reversed-PE images to the 3D displacement field. *Box*: output feature maps of a layer. *Number inside each box*: number of feature maps in the layer. *Number below each box*: feature map size relative to the full input image size.

The deep convolutional network can be considered as a mapping function $f_{\theta }:\left(I_{1},I_{2}\right)\to U$, where θ is the set of network parameters. The deep network, which is inspired by S-Net [16], U-Net [23], and DL-GP [24], is U-Net-like architecture with an encoder and a decoder (see Figure 2). The encoder takes a two-channel input (which is the reverse PE image pair) and extracts the latent features. The decoder takes the latent features to predict the displacement field.

Both the encoder and the decoder use a kernel size of 3×3×3 voxels for their convolutional layers to extract information from the neighboring voxels. This kernel size is selected because it requires fewer trainable parameters than larger kernel sizes, thereby improving computational efficiency. Each convolutional layer is followed by a BN layer to mitigate changes in the distribution of the convolutional layer’s input [25].

To make TS-Net cope with different input image sizes, we add a size-normalization layer before the encoder and a size-recovery layer after the decoder. The size-normalization layer uses zero-padding so that each input dimension is divisible by 16. The size-recovery layer crops the decoder output to the size of the input image. To resize images, TS-Net uses zero-padding instead of interpolation to maintain the spatial resolution of the input images. Maintaining the original spatial resolution is critical in SAC because the displacements in the EPI images are small and sensitive to image interpolation. Note that the configuration of the introduced convolutional encoder–decoder, e.g. the number of layers, batch normalization, and upsampling layers, was experimentally selected, see Section 3.1.

In our previous deep-learning-based SAC method [16], the network parameters θ are estimated by optimizing the objective function that promotes the similarity between the pair of corrected images and enforces the local smoothness of the predicted displacement field. In this study, we regularize the training by introducing a $T_{1w}$-based regularizer to the loss function. This regularizer can improve the TS-Net training as the $T_{1w}$ image is widely considered a gold standard representation of a subject’s brain anatomy [17]. Note that $T_{1w}$ images are used in the training phase, not in the testing phase.

The $T_{1w}$-based regularizer penalizes the distances from the corrected images to the corresponding $T_{1w}$ structural image. Since $T_{1w}$ and EPI are in different modalities, we use the normalized mutual information (NMI) to measure the similarity between the output images and the $T_{1w}$ image because it is effective for multi-modal images. Let *A* denote the $T_{1w}$ image, and the $T_{1w}$-based regularizer is then defined as

(1) $$L_{\mathrm{anat}}\left(E_{1},\;E_{2},\;A\right)=1\;-\;\frac{\mathrm{NMI}\left(E_{1},\;A\right)+\mathrm{NMI}\left(E_{2},\;A\right)}{2}.$$

The loss for TS-Net training is

(2) $$\begin{array}{cc} L(I_{1},\;I_{2},\;A,\;U)=\; & L_{\mathrm{sim}}\left(E_{1},\;E_{2}\right)\;+\;\lambda L_{\mathrm{smooth}}\left(U\right)\;+\gamma L_{\mathrm{anat}}\left(E_{1},\;E_{2},\;A\right), \end{array}$$

where $L_{\mathrm{sim}}$ is the dissimilarity between the pair of corrected images. $L_{\mathrm{smooth}}$ is the diffusion regularizer, denoting the non-smoothness of the predicted displacement field. The positive and user-defined regularization parameters λ and γ represent the trade-off between the similarity of the corrected images, the smoothness of the displacement field, and the similarity of the $T_{1w}$ image to the output images.

Since the corrected images $E_{1}$ and $E_{2}$ have the same modality, we investigate three possible unimodal similarity metrics: mean squared error (MSE), local cross-correlation (LCC) [26], and local normalized cross-correlation (LNCC) [27] (refer to Appendix A.1 for a detailed description of the metrics). We experimentally found that LNCC metric is the best choice in terms of the trade-off between training accuracy and processing time (see the analysis in Section 3.1). Thus, LNCC is used as the $L_{\mathrm{sim}}$.

### 2.3. Experimental Methods

To evaluate TS-Net, for each dataset, we first split the subjects randomly into two parts: A and B. Then, the training set was formed by randomly selecting reversed-PE image pairs of each subject in Part A; this strategy reduces the data repetition of subjects. The test set was formed from all reversed-PE pairs of each subject in Part B. The training sets were used to select the hyper-parameters and train the TS-Net models, and the test sets were used to evaluate the correction accuracy of the TS-Net models. The training set of each dataset was further divided into a training set and a validation set with a ratio of 9:1. Table 2 summarizes the training, validation, and test sets of the four datasets.

The proposed TS-Net was implemented using Keras [28] deep learning library. For training TS-Net, the Adam optimizer was used with the learning rate α=0.001 and the exponential decay rates $\beta_{1}=0.9$ and $\beta_{2}=0.999$, as suggested by Kingma and Ba [29]. The Tree of Parzen Estimator algorithm was used to select suitable values for regularization parameters λ and γ [30,31,32]. In training each dataset, we selected the maximum batch size that could fit into the available GPU memory to reduce the training time. The batch sizes and regularization parameters used in training TS-Net are shown in Table 3.

We then compared the proposed TS-Net with two iterative-optimization methods, i.e., TOPUP and TISAC, and a state-of-the-art deep learning method, i.e., S-Net. The comparison is in terms of the correction accuracy and processing speed. To evaluate the correction accuracy of the proposed method, we trained S-Net and TS-Net for 1500 epochs with each dataset. The trained models were used to compute the corrected image pairs of the test sets. For TOPUP (we used the TOPUP implementation in the FSL package, website: fsl.fmrib.ox.ac.uk/fsl/fslwiki/topup, accessed on 1 May 2020) and TISAC, the corrected image pairs were obtained by implementing the iterative-optimization algorithms. Here, the correction accuracy is measured in terms of LNCC similarity between the pair of reversed-PE images.

The experiments were conducted using images from the datasets directly, without any pre-processing step. The experiments for evaluating processing times were performed on a system that has an Intel Core i5-9600K CPU at 3.6 GHz, 32 GB of RAM, and an NVIDIA GeForce RTX2080 GPU with 8 GB memory. The other experiments were performed on a system that has an Intel Xero Gold 5115 CPU at 2.4 GHz and an NVIDIA GeForce GTX Titan Xp with 12 GB memory.

## 3. Results

In this section, Section 3.1 presents the results of the ablation study. Section 3.2 shows the results of the proposed method and other representative SAC methods in terms of correction accuracy and processing time.

### 3.1. Ablation Study of the Proposed Method

This section analyzes the proposed TS-Net method in five aspects: (i) the effects of using different similarity measures; (ii) the effects of the different network configurations in TS-Net; (iii) the effects of using the 3D distortion model and $T_{1w}$ regularization; (iv) the effects of using a pre-trained TS-Net in training other datasets; (v) the visualization of the predicted displacement field.

**Figure 3:**
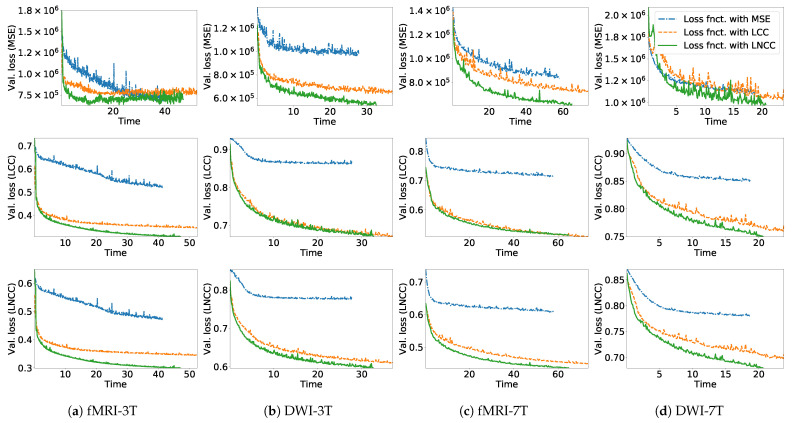
Validation loss of the models trained with three types of similarity loss (MSE, LCC, and LNCC) versus training time (in hours) on the four datasets: (**a**) fMRI-3T; (**b**) DWI-3T; (**c**) fMRI-7T; (**d**) DWI-7T. *Top row*: validation loss in terms of MSE. *Middle row*: validation loss in terms of LCC. *Bottom row*: validation loss in terms of LNCC.

**Effects of similarity measures in network training:** In this experiment, for each training set, we trained TS-Net models using different similarity losses: (i) MSE; (ii) LCC; (iii) LNCC. The effects of using different similarity measures were evaluated in two aspects: the validation loss and the training time of each epoch. The validation loss was measured as the mean similarity measures for output image pairs across subsets of the training sets. We conducted experiments on the four datasets: fMRI-3T, DWI-3T, fMRI-7T, and DWI-7T. Figure 3 shows the validation loss versus time when training TS-Net with the similarity loss as MSE, LCC, and LNCC. It can be seen that TS-Net trained with the LNCC measure produces the lowest validation loss, while TS-Net trained with the MSE measure produces the highest validation loss. TS-Nets trained with the LNCC and LCC measures produce a competitive LCC validation loss on two datasets (DWI-3T and fMRI-7T). Considering the validation loss versus the training time, it is clear that the LNCC measure is a better choice than the MSE and the LCC for training TS-Net. Based on this experiment, the LNCC metric was subsequently used as the similarity loss for all the remaining experiments.

**Effects of the network configurations in TS-Net:** In this experiment, we analyzed the effects of four different network configurations: (i) TS-Net without batch normalization and with an upsampling layer (UL); (ii) TS-Net with instance normalization (IN) [33] and with UL; (iii) TS-Net with BN and transposed convolution (TC) [34]; (iv) TS-Net with BN and UL (the proposed method). The validation loss during the training phase was computed as the average LNCC measure between the output image pairs, across subsets of the training sets. This validation loss was then used to compare different network configurations.

**Figure 4:**
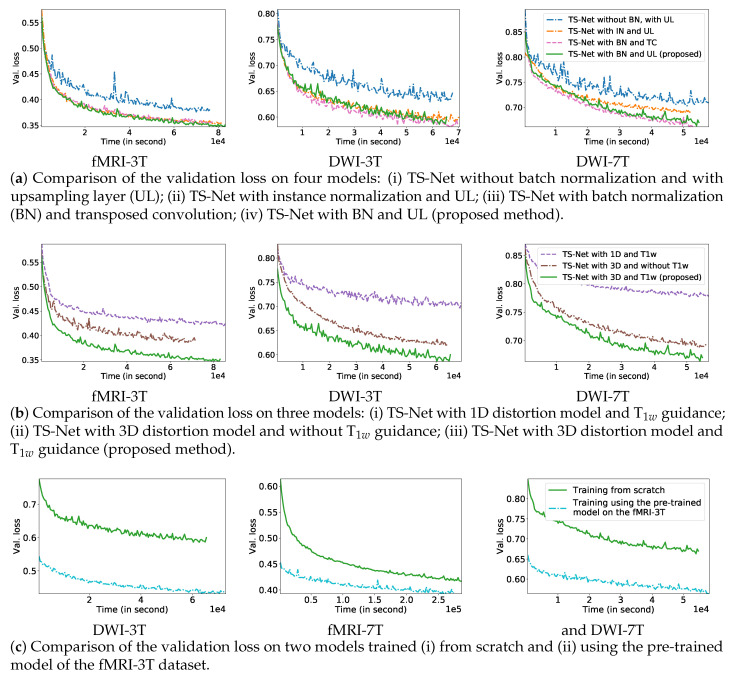
Ablation study of TS-Net in terms of (**a**) network configurations, (**b**) the 3D distortion model and anatomical guidance, and (**c**) using a pre-trained model. Plots show the validation loss of trained models versus training time (in seconds).

Figure 4a shows the validation loss versus the training time on three datasets: fMRI-3T, DWI-3T, and DWI-7T; each subfigure includes the validation loss for the four network configurations. Several observations can be made. First, using batch normalization (the proposed TS-Net, green curve) provides a lower validation loss compared to not using batch normalization (blue curve). Second, using batch normalization (the proposed TS-Net, green curve) provides a similar or lower validation loss compared to using instance normalization (orange curve). Third, using the upsampling layer (the proposed TS-Net, green curve) has a similar validation loss compared to using the transpose convolution (magenta curve). These results justify our selected configuration for TS-Net.

**Effects of using the 3D distortion model and anatomical guidance by $T_{1w}$:** In this experiment, we trained three types of networks: (i) TS-Net with the 1D distortion model as used in S-Net [16]; (ii) TS-Net with the 3D distortion model and without $T_{1w}$ guidance; (iii) TS-Net with the 3D distortion model and $T_{1w}$ guidance (the proposed method). Figure 4b shows the validation loss versus the training time on three datasets: fMRI-3T, DWI-3T, and DWI-7T. Several observations can be made. First, the proposed TS-Net with $T_{1w}$ guidance (green solid curve) has lower validation losses than the TS-Net without $T_{1w}$ guidance (brown dash-dotted curve). This result shows that incorporating $T_{1w}$ guidance can improve the correction accuracy. Second, the proposed TS-Net using the 3D distortion model (green solid curve) produces significantly lower validation losses than TS-Net using the 1D distortion model (magenta dashed curve). This result shows that the 3D distortion model used in the proposed TS-Net provides more accurate correction than the 1D distortion model (i.e., only along the phase-encoding direction), which is used in S-Net and existing iterative-optimization SAC methods.

**Effects of using a pre-trained TS-Net:** In this experiment, we explored whether using a TS-Net model pre-trained on one dataset can reduce the training time on another dataset, compared to a randomly initialized TS-Net. To this end, we trained two TS-Net models: (i) from scratch; (ii) using an *initial* network, which had been pre-trained for 1500 epochs on the fMRI-3T dataset. Figure 4c shows the validation loss versus training time on three datasets: DWI-3T, fMRI-7T, and DWI-7T. The figure shows that the validation loss when training TS-Net using a pre-trained model (cyan dash-dotted curve) is much lower than when training from scratch (green solid curve). The result suggests that TS-Net is able to learn generalized features for correcting the susceptibility artifacts from one dataset. Subsequently, adopting the learned features in training other datasets leads to a faster converge.

**Figure 5:**
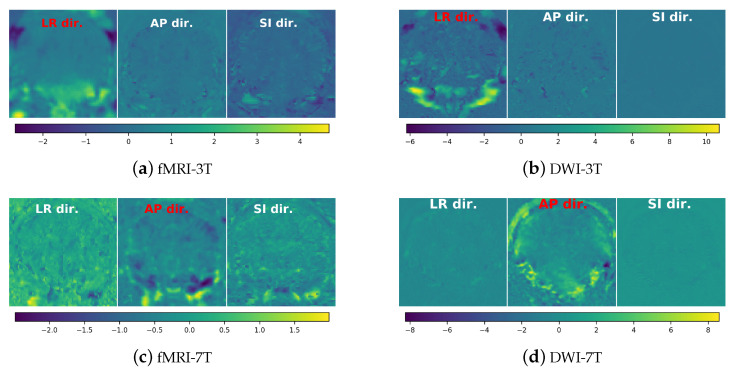
Samples of three predicted displacement fields (in voxel) of TS-Net from the four test sets. In each subfigure, *left image*: displacement field in the left–right (LR) direction; *middle image*: displacement field in the anterior–posterior (AP) direction; *right image* displacement field in the superior–inferior (SI) direction. The dominant phase-encoding dimension (direction) is shown in red text; the other two other dimensions are shown in white text.

**Visualization of the predicted displacement fields:**Figure 5 shows the samples of the displacement field estimated by the trained TS-Net for the four test sets. The displacement field is shown in three directions (left–right, anterior–posterior, and superior–inferior). TS-Net can estimate the geometric distortions along the directions that are not the dominant PE direction. The visual results indicate that TS-Net is able to predict realistic 3D displacement fields, i.e., the displacements in the phase-encoding direction are more dominant than those in the other two directions.

### 3.2. Comparison with Other Methods

**Figure 6:**
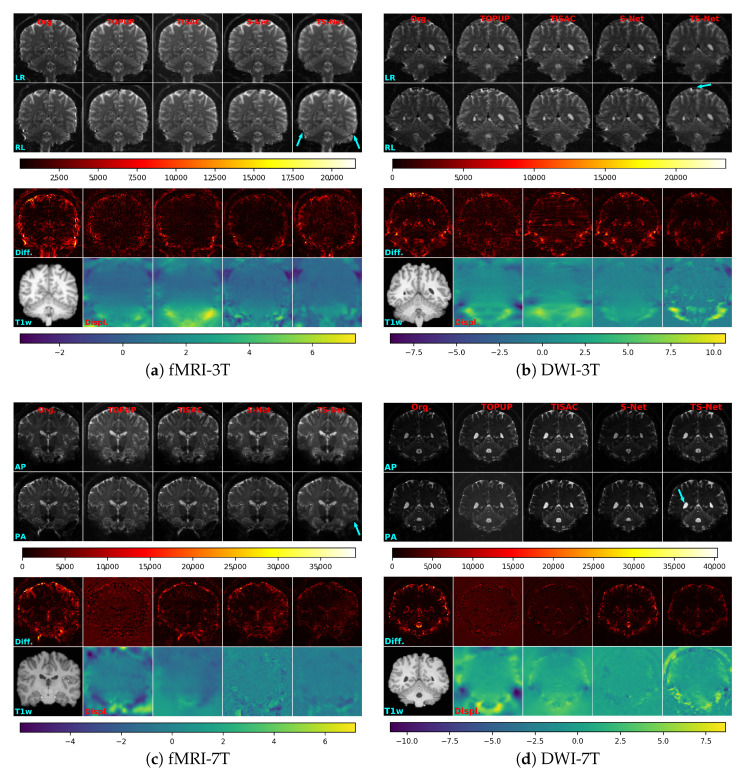
Sample visual results of SAC methods from the four test sets. In each subfigure, *Column 1*: input uncorrected images. *Columns 2, 3, 4, and 5*: output corrected images produced by TOPUP, TISAC, S-Net, and TS-Net, respectively. *Rows 1 and 2*: reversed phase-encoding EPI images. *Row 3*: the color bar of the absolute different maps. *Row 4*: the absolute difference between the image pair. *Row 5*: the corresponding $T_{1w}$ image of the reversed-PE images and the estimated displacement fields of the compared SAC methods. For visualization, only the displacement field in the phase-encoding direction of TS-Net is shown. *Row 6*: the color bar of the displacement fields, in which the number expresses the number of voxels shifted.

**Figure A1:**
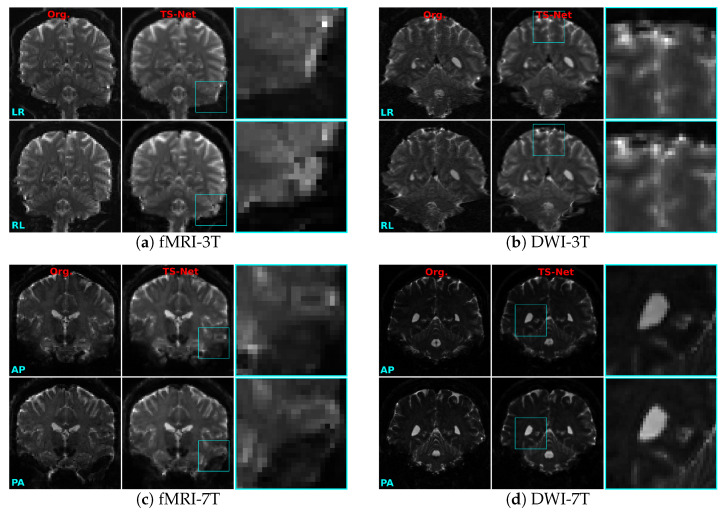
Larger view of the TS-Net outputs from the four test sets. In each subfigure, *Column 1*: input uncorrected images. *Column 2*: output corrected images produced by TS-Net. *Column 3*: the zoomed view of cyan rectangles from the TS-Net output.

This section compares TS-Net with three SAC methods, i.e., TOPUP, TISAC, and S-Net. Figure 6 shows sample slices of uncorrected and corrected images from each of the four test sets. Each example includes two reversed-PE images (Rows 1 and 2) and the absolute difference between the two images (Row 3). The arrows indicate the regions where TS-Net produces significantly improved correction in comparison with three other SAC methods. It can be seen that TS-Net removes distortions in the uncorrected images significantly. In general, TS-Net produces the output images that are comparable to or better than the outputs of TOPUP, TISAC, and S-Net. Note that the SAC methods work with 3D images; however, for visualization, 2D slices are presented in the figures. For a larger view of the TS-Net outputs, see Figure A1 in Appendix B.

Table 4 summarizes the accuracy of uncorrected and corrected images in terms of LNCC on four different test sets. Paired t-tests were performed on the LNCC measures between TS-Net outputs and each of four image types: uncorrected images, TOPUP outputs, TISAC outputs, and S-Net outputs. The null hypothesis is $H_{0}:m_{S-\mathrm{Net}}=m_{\mathrm{other}}$. All computed *P* values are smaller than 0.001; this indicates that the null hypothesis is rejected at a confidence level of 99.9%. In other words, TS-Net produces image pairs with significant differences (i.e., improvements) in terms of accuracy compared to the output image pairs of other methods.

**Figure 7:**
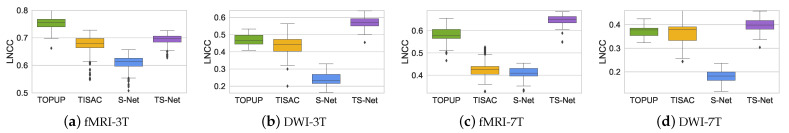
Comparisons of the proposed TS-Net versus other three SAC methods in terms of the LNCC-based accuracy on the test sets. Due to differences in the LNCC ranges of the datasets, the plots are drawn in different *y*-axis ranges for clarity. In each box plot, the *top line* is the maximum LNCC value excluding the outliers; the *bottom line* is the minimum LNCC value excluding the outliers; the *middle line* is the median LNCC value; the *solid rectangle* is the interquartile range of the LNCC values; the *points* are the outliers.

For visual clarity, Figure 7 shows the box plots for comparing the LNCC measures of the four SAC methods. The results in Table 4 and Figure 7 show three notable observations. First, TS-Net produces output images that have significantly higher LNCC measures than the uncorrected images; in other words, TS-Net does reduce the susceptibility artifacts. Second, TS-Net produces output images that have higher LNCC measures than the outputs of the TISAC method in four out of four datasets, and the outputs of the TOPUP methods in three out of four datasets. This means that TS-Net has better correction accuracy compared to the two iterative-optimization methods, i.e., TISAC and TOPUP. Third, TS-Net also produces higher LNCC measures than S-Net in four out of four datasets. Compared to S-Net, the proposed TS-Net has several differences, one of which is its use of $T_{1w}$ images in training. This result demonstrates that including the *gold-standard* representation of a subject’s brain anatomy helps regularize the susceptibility artifact correction in TS-Net. Note that TS-Net does not require the $T_{1w}$ image in the inference phase, which explains its comparable processing speed with S-Net, as analyzed next.

To compare the processing speed, we first randomly selected 50 distorted image pairs for each of the four datasets. We then recorded the time for correcting the selected image pairs by four SAC methods: TOPUP, TISAC, S-Net, and TS-Net. Table 5 shows the average processing time per image pair of TS-Net and the three SAC methods. Over the four datasets, TS-Net is 396.72 times faster than TOPUP, 29.45 times faster than TISAC, and only 1.05 times slower than S-Net. Both deep learning-based SAC methods (TS-Net and S-Net) can be accelerated by five times when using the GPU instead of the CPU. Note that, in the experiments for all datasets, the proposed TS-Net has 260,187 trainable parameters, whereas the S-Net model has 259,241 trainable parameters. In other words, the proposed TS-Net requires only 0.36% more trainable parameters than S-Net.

The results of TS-Net over the four datasets show that the inference time of TS-Net is linearly proportional to the size of the input images. To correct an image pair with a size of 90 × 104 × 72, TS-Net takes 0.65 s using CPU and 0.14 s using GPU. On average, the inference speed of TS-Net is approximately 1.08 million voxels per second with CPU and 5.98 million voxels per second with GPU.

## 4. Discussion

This section discusses the proposed TS-Net in three aspects: robustness, generalizability, and feasibility. In terms of robustness, TS-Net can predict realistic 3D displacement fields, i.e., the most dominant displacements in the phase-encoding direction regardless of the PE direction order, resulting in high-quality corrected images. The experiments conducted on four different datasets show that TS-Net performed consistently on different image resolutions, image sizes, image modalities, and training set sizes. Furthermore, it can cope with different phase-encoding directions.

In terms of generalizability, TS-Net is able to learn the generalized features of the susceptibility artifacts in reversed-PE image pairs from one dataset. A trained TS-Net can be easily transferred to a new dataset, effectively reducing the training time. This observation is similar to the generalization capability of the deep networks [35]. Therefore, TS-Net can employ the network initialization techniques, e.g. MAML [36] and Reptile [37], to address the problem of a long training time, which is a common bottleneck in deep learning algorithms.

In terms of feasibility, TS-Net can produce higher accuracy than the state-of-the-art SAC methods, while having a fast processing time. To correct a pair of distorted images, TS-Net only takes less than 5 s using CPU or less than 1 s using GPU. These high-accuracy and high-speed capabilities allow TS-Net to be applied in many applications. For example, TS-Net can be integrated into the MRI scanner to correct SAs in real time; this is typically not possible with the traditional reversed-PE SAC methods because they are slow.

## 5. Conclusions

This paper presents an end-to-end 3D anatomy-guided deep learning framework, TS-Net, to correct the susceptibility artifacts in reversed phase-encoding 3D EPI image pairs. The proposed TS-Net contains a deep convolutional network to predict the displacement field in all three directions. The corrected images are then generated by feeding the predicted displacement field and input images into a 3D spatial transform unit. In the training phase, the proposed TS-Net additionally utilizes $T_{1w}$ images to regularize the susceptibility artifact correction. However, the $T_{1w}$ images are not used in the inference phase to simplify the use of TS-Net.

The visual analysis shows that TS-Net is able to estimate the realistic 3D displacement field, i.e., the displacements are dominant in the phase-encoding direction, compared with the other two directions. Evaluation on the four large datasets also demonstrates that the proposed TS-Net provides higher correction accuracy than TISAC and S-Net in all four datasets, and TOPUP in three out of four datasets. Over the four datasets, TS-Net runs significantly faster than the iterative-optimization SAC methods: 396.72 times faster than TOPUP and 29.45 times faster than TISAC. TS-Net is slightly slower than S-Net, but it still meets the real-time correction requirement of MRI scanners. Furthermore, the training time of TS-Net on a new dataset can be reduced by using a pre-trained model.

## Figures and Tables

**Table 1:** A summary of the datasets used in the experiments.

Datasets	No. Subjs.	Gender Distribution		Age Distribution		Image Size (Voxels)	Resolution (mm${}^{3}$)	Acquisition Sequences	BW Hz/P${}_{x}$	Field Strength	PE Directions
**fMRI-3T**	182	Males:	72	22–25 years:	24	90 × 104 × 72	2 × 2 × 2	Multi-band 2D gradient-echo EPI, factor of 8	2290	3T	LR and RL
				26–30 years:	85						
		Females:	110	31–35 years:	71						
				over 36 years:	2						
**DWI-3T**	180	Males:	71	22–25 years:	23	144 × 168 × 111	1.25 × 1.25 × 1.25	Multi-band 2D spin-echo EPI, factor of 3	1488	3T	LR and RL
				26–30 years:	84						
		Females:	109	31–35 years:	71						
				over 36 years:	2						
**fMRI-7T**	184	Males:	72	22–25 years:	24	130 × 130 × 85	1.6 × 1.6 × 1.6	Multi-band 2D gradient-echo EPI, factor of 5	1924	7T	AP and PA
				26–30 years:	85						
		Females:	112	31–35 years:	73						
				over 36 years:	2						
**DWI-7T**	178	Males:	69	22–25 years:	21	200 × 200 × 132	1.05 × 1.05 × 1.05	Multi-band 2D spin-echo EPI, factor of 2	1388	7T	AP and PA
				26–30 years:	85						
		Females:	109	31–35 years:	70						
				over 36 years:	2						

Abbreviations: BW = Readout bandwidth; LR = left-to-right; RL = right-to-left; AP = anterior-to-posterior; PA = posterior-anterior.

**Table 2:** A summary of the training, validation, and test sets for each of the four datasets.

Datasets	Training Set		Validation Set		Test Set	
	No. Subjects	No. Pairs	No. Subjects	No. Pairs	No. Subjects	No. Pairs
**fMRI-3T**	140	1685	16	187	26	1395
**DWI-3T**	135	392	15	44	30	90
**fMRI-7T**	138	2890	15	322	31	1269
**DWI-7T**	133	140	15	15	30	60

**Table 3:** Values of hyper-parameters in training TS-Net on the four datasets.

Params	fMRI-3T	DWI-3T	fMRI-7T	DWI-7T
λ	0.1771	0.002	0.9323	0.025
γ	0.01	0.01	0.01	0.01
Batch size	4	1	1	1

**Table 4:** Accuracy in terms of local normalized cross-correlation for different test sets: fMRI-3T, DWI-3T, fMRI-7T, and DWI-7T. The best measurements are given in bold.

Datatypes	fMRI-3T	DWI-3T	fMRI-7T	DWI-7T
	mean ± std	mean ± std	mean ± std	mean ± std
Uncorrected	0.335 * ± 0.023	0.142 * ± 0.020	0.229 * ± 0.023	0.120 * ± 0.018
TOPUP	**0.753** * ± 0.024	0.468 * ± 0.031	0.583 * ± 0.024	0.371 * ± 0.025
TISAC	0.674 * ± 0.036	0.436 * ± 0.058	0.427 * ± 0.036	0.364 * ± 0.048
S-Net	0.608 * ± 0.027	0.242 * ± 0.039	0.412 * ± 0.027	0.182 * ± 0.025
TS-Net	0.692 ± 0.022	**0.571** ± 0.034	**0.648** ± 0.022	**0.398** ± 0.031

The asterisk symbol (*) indicates that the computed *p* is less than 0.001 for the null hypothesis $H_{0}:m_{S-\mathrm{Net}}=m_{\mathrm{other}}$. A *p* value below 0.001 means that the null hypothesis is rejected at a confidence level of 99.9%. In other words, the similarity measure LNCC of TS-Net is significantly different from the compared method.

**Table 5:** Processing time (in seconds) of SAC methods for correcting a pair of reversed-PE images.

Methods	Processor	fMRI-3T 90 × 104 × 72	DWI-3T 144 × 168 × 111	fMRI-7T 130 × 130 × 85	DWI-7T200 × 200 × 132
		(Mean ± std)	(Mean ± std)	(Mean ± std)	(Mean ± std)
TOPUP	CPU	252.55 ± 3.61	997.39 ± 9.04	535.71 ± 44.29	1944.65 ± 18.72
TISAC		25.76 ± 11.81	57.73 ± 12.03	28.48 ± 5.14	126.13 ± 26.25
S-Net		0.63 ± 0.03	2.21 ± 0.03	1.36 ± 0.03	4.55 ± 0.04
TS-Net		0.65 ± 0.04	2.30 ± 0.05	1.45 ± 0.04	4.92 ± 0.06
S-Net	GPU	0.13 ± 0.14	0.42 ± 0.18	0.22 ± 0.16	0.72 ± 0.25
TS-Net		0.14 ± 0.16	0.43 ± 0.21	0.23 ± 0.18	0.80 ± 0.26

## Data Availability

This research used the EPI data provided by the Human Connectome Project, which can be accessed via https://www.humanconnectome.org/study/hcp-young-adult/document/1200-subjects-data-release (accessed on 11 November 2019), with appropriate data usage agreement.

## Appendix A. Similarity Metrics

This section presents the three similarity metrics, i.e., MSE, LCC, and LNCC, which are used in $L_{\mathrm{sim}}$.

### Appendix A.1. Mean Squared Error

The mean squared error between two images $E_{1}$ and $E_{2}$ is defined as

(A1) $$\mathrm{MSE}\left(E_{1},\;E_{2}\right)=\frac{1}{\left|\Omega \right|}\sum_{p\in \Omega }{\left[E_{1}\left(p\right)\;-\;E\left(p\right)\right]}^{2},$$

where $\Omega \in R^{3}$ is the image domain, and |Ω| is the total number of image indexes. A smaller value of MSE indicates a higher similarity between the images. Thus, the $L_{\mathrm{sim}}$ loss based on the MSE measure is

(A2) $$L_{\mathrm{sim}}^{\mathrm{MSE}}\left(E_{1},\;E_{2}\right)=\mathrm{MSE}\left(E_{1},\;E_{2}\right).$$

### Appendix A.2. Local Cross-Correlation

The local cross-correlation metric can be explained as follows. Consider an image *X*. Let $\bar{X}$ be the local mean image obtained by applying an n×n×n averaging filter on *X*. The local centered image $\hat{X}$ is computed as

(A3) $$\hat{X}=X-\bar{X}.$$

For a given voxel p=(x,y,z), let W(p) denote the set of voxels in the n×n×n cube centered on p. For a pair of images $E_{1}$ and $E_{2}$, we compute a local correlation coefficient image *C*:

(A4) $$C\left(p\right)=\frac{{\left(\sum_{p_{i}\in W\left(p\right)}\left[{\hat{E}}_{1}\left(p_{i}\right)\;{\hat{E}}_{2}\left(p_{i}\right)\right]\right)}^{2}}{\sum_{p_{i}\in W\left(p\right)}{\left[{\hat{E}}_{1}\left(p_{i}\right)\right]}^{2}\;\sum_{p_{i}\in W\left(p\right)}{\left[{\hat{E}}_{2}\left(p_{i}\right)\right]}^{2}}.$$

The LCC measure for images $E_{1}$ and $E_{2}$ is now defined as the mean intensity of the local correlation image *C*:

(A5) $$\mathrm{LCC}\left(E_{1},\;E_{2}\right)=\frac{1}{\left|\Omega \right|}\sum_{p\in \Omega }C\left(p\right).$$

A higher LCC indicates more similarity between two output images. We now can express the $L_{\mathrm{sim}}$ loss based on the LCC measure as

(A6) $$L_{\mathrm{sim}}^{\mathrm{LCC}}\left(E_{1},\;E_{2}\right)=1-\mathrm{LCC}\left(E_{1},\;E_{2}\right).$$

### Appendix A.3. Local Normalized Cross-Correlation

The local normalized cross-correlation metric can be defined as follows. Let $\tilde{X}$ be the variance image of *X*:

(A7) $$\tilde{X}\left(p\right)=\sum_{p_{i}\in W\left(p\right)}{\left[X\left(p_{i}\right)\right]}^{2}\;-\;\frac{1}{n^{3}}{\left[\sum_{p_{i}\in W\left(p\right)}X\left(p_{i}\right)\right]}^{2}.$$

Let *R* be the correlation image between two images $E_{1}$ and $E_{2}$:

(A8) $$\begin{array}{cc} R(p)= & \sum_{p_{i}\in W\left(p\right)}\left[E_{1}\left(p_{i}\right)\;E_{2}\left(p_{i}\right)\right]\;-\;\frac{1}{n^{3}}\sum_{p_{i}\in W\left(p\right)}E_{1}\left(p_{i}\right)\;\sum_{p_{i}\in W\left(p\right)}E_{2}\left(p_{i}\right). \end{array}$$

The LNCC between two images $E_{1}$ and $E_{2}$ is given by

(A9) $$\mathrm{LNCC}\left(E_{1},\;E_{2}\right)=\frac{1}{\left|\Omega \right|}\sum_{p\in \Omega }\frac{{\left[R\left(p\right)\right]}^{2}}{{\tilde{E}}_{1}\left(p\right)\;{\tilde{E}}_{2}\left(p\right)}.$$

A higher LNCC indicates higher similarity between two output images. We now can express the $L_{\mathrm{sim}}$ loss based on the LNCC measure as

(A10) $$L_{\mathrm{sim}}^{\mathrm{LNCC}}\left(E_{1},\;E_{2}\right)=1-\mathrm{LNCC}\left(E_{1},\;E_{2}\right).$$

## References

[1] Poustchi-Amin M., Mirowitz S.A., Brown J.J., McKinstry R.C., Li T. (2001). Principles and applications of echo-planar imaging: A review for the general radiologist. Radiographics.

[2] Matthews P.M., Honey G.D., Bullmore E.T. (2006). Applications of fMRI in translational medicine and clinical practice. Nat. Rev. Neurosci..

[3] Baars B.J., Gage N.M. (2013). Brain imaging. Fundamentals of Cognitive Neuroscience.

[4] Chang H., Fitzpatrick J.M. (1992). A technique for accurate magnetic resonance imaging in the presence of field inhomogeneities. IEEE Trans. Image Process..

[5] Schmitt F. (2015). Echo-Planar Imaging. Brain Mapping—An Encyclopedic Reference.

[6] Chan R.W., von Deuster C., Giese D., Stoeck C.T., Harmer J., Aitken A.P., Atkinson D., Kozerke S. (2014). Characterization and correction of Eddy-current artifacts in unipolar and bipolar diffusion sequences using magnetic field monitoring. J. Magn. Reson..

[7] Irfanoglu M.O., Sarlls J., Nayak A., Pierpaoli C. (2019). Evaluating corrections for Eddy-currents and other EPI distortions in diffusion MRI: Methodology and a dataset for benchmarking. Magn. Reson. Med..

[8] Jezzard P., Balaban R.S. (1995). Correction for geometric distortion in echo planar images from B0 field variations. Magn. Reson. Med..

[9] Holland D., Kuperman J.M., Dale A.M. (2010). Efficient correction of inhomogeneous static magnetic field-induced distortion in echo planar imaging. NeuroImage.

[10] Andersson J.L.R., Skare S., Ashburner J. (2003). How to correct susceptibility distortions in spin-echo echo-planar images: Application to diffusion tensor imaging. NeuroImage.

[11] Ruthotto L., Kugel H., Olesch J., Fischer B., Modersitzki J., Burger M., Wolters C.H. (2012). Diffeomorphic susceptibility artifact correction of diffusion-weighted magnetic resonance images. Phys. Med. Biol..

[12] Hedouin R., Commowick O., Bannier E., Scherrer B., Taquet M., Warfield S.K., Barillot C. (2017). Block-matching distortion correction of echo-planar images with opposite phase encoding directions. IEEE Trans. Med. Imaging.

[13] Irfanoglu M.O., Modia P., Nayaka A., Hutchinson E.B., Sarllsc J., Pierpaoli C. (2015). DR-BUDDI (diffeomorphic registration for blip-up blip-down diffusion imaging) method for correcting echo planar imaging distortions. NeuroImage.

[14] Duong S.T.M., Schira M.M., Phung S.L., Bouzerdoum A., Taylor H.G.B. Anatomy-guided inverse-gradient susceptibility artefact correction method for high-resolution fMRI. Proceedings of the 2018 IEEE International Conference on Acoustics, Speech and Signal Processing (ICASSP).

[15] Duong S.T.M., Phung S.L., Bouzerdoum A., Taylor H.G.B., Puckett A.M., Schira M.M. (2020). Susceptibility artifact correction for sub-millimeter fMRI using inverse phase encoding registration and T1 weighted regularization. J. Neurosci. Methods.

[16] Duong S.T.M., Phung S.L., Bouzerdoum A., Schira M.M. (2020). An unsupervised deep learning technique for susceptibility artifact correction in reversed phase-encoding EPI mages. Magn. Reson. Imaging.

[17] Howarth C., Hutton C., Deichmann R. (2006). Improvement of the image quality of T1-weighted anatomical brain scans. NeuroImage.

[18] Polimeni J.R., Renvall V., Zaretskaya N., Fischl B. (2018). Analysis strategies for high-resolution UHF-fMRI data. NeuroImage.

[19] Essen D.C.V., Ugurbil K., Auerbach E., Barch D., Behrens T.E.J., Bucholz R., Chang A., Chen L., Corbetta M., Curtiss S.W. (2012). The human connectome project: A data acquisition perspective. NeuroImage.

[20] Essen D.C.V., Smith S.M., Barch D.M., Behrens T.E.J., Yacoub E., Ugurbil K. (2013). The WU-Minn human connectome project: An overview. NeuroImage.

[21] Ugurbil K., Xu J., Auerbach E.J., Moeller S., Vu A.T., Duarte-Carvajalino J.M., Lenglet C., Wu X., Schmitter S., Moortele P.F.V.D. (2013). Pushing spatial and temporal resolution for functional and diffusion MRI in the Human Connectome Project. NeuroImage.

[22] Balakrishnan G., Zhao A., Sabuncu M.R., Guttag J., Dalca A.V. (2019). VoxelMorph: A learning framework for deformable medical image registration. IEEE Trans. Med. Imaging.

[23] Ronneberger O., Fischer P., Brox T. (2015). U-Net: Convolutional networks for biomedical image segmentation. International Conference on Medical Image Computing and Computer-Assisted Intervention.

[24] Nguyen T.N.A., Phung S.L., Bouzerdoum A. (2020). Hybrid deep learning-Gaussian process network for pedestrian lane detection in unstructured scenes. IEEE Trans. Neural Netw. Learn. Syst..

[25] Ioffe S., Szegedy C. Batch normalization: Accelerating deep network training by reducing internal covariate shift. Proceedings of the International Conference on Machine Learning.

[26] Avants B.B., Epstein C.L., Grossman M., Gee J.C. (2008). Symmetric diffeomorphic image registration with cross-correlation: Evaluating automated labeling of elderly and neurodegenerative brain. Med. Image Anal..

[27] Baig A., Chaudhry M.A., Mahmood A. Local normalized cross correlation for geo-registration. Proceedings of the 2012 9th International Bhurban Conference on Applied Sciences & Technology (IBCAST).

[28] Chollet F. Keras. https://github.com/fchollet/keras.

[29] Kingma D., Ba J. (2014). Adam: A method for stochastic optimization. arXiv.

[30] Bergstra J., Bardenet R., Bengio Y., Kegl B. Algorithms for hyper-parameter optimization. Proceedings of the 25th Annual Conference on Neural Information Processing Systems (NIPS 2011).

[31] Bergstra J., Yamins D., Cox D.D. Making a science of model search: Hyperparameter optimization in hundreds of dimensions for vision architectures. Proceedings of the International Conference on Machine Learning.

[32] Bergstra J., Komer B., Eliasmith C., Yamins D., Cox D.D. (2015). Hyperopt: A Python library for model selection and hyperparameter optimization. Comput. Sci. Discov..

[33] Ulyanov D., Vedaldi A., Lempitsky V. (2016). Instance normalization: The missing ingredient for fast stylization. arXiv.

[34] Long J., Shelhamer E., Darrell T. Fully convolutional networks for semantic segmentation. Proceedings of the IEEE Conference on Computer Vision and Pattern Recognition.

[35] Neyshabur B., Bhojanapalli S., McAllester D., Srebro N. Exploring generalization in deep learning. Proceedings of the International Conference on Neural Information Processing Systems.

[36] Finn C., Abbeel P., Levine S. Model-agnostic meta-learning for fast adaptation of deep networks. Proceedings of the International Conference on Machine Learning.

[37] Nichol A., Achiam J., Schulman J. (2018). On first-order meta-learning algorithms. arXiv.

